# On the Treatment of the Commoner Forms of Skin Diseases

**Published:** 1883-06-20

**Authors:** J. Leslie Foley

**Affiliations:** M. D., C. M., L.R.C.P. London; Assistant Demonstrator of Anatomy, Faculty of Medicine, Bishop’s College, Attending Physician to the Montreal Dispensary


### ON THE TREATMENT OF THE COMMONER FORMS OF SKIN DISEASES.

By J. LESLIE FOLEY, M. D., C. M., L. R. C. P., LONDON,
Assistant Demonstrator of Anatomy, Faculty of Medicine, Bishop's College, Attending Physician to the Montreal Dispensary.

To arrive at a correct diagnosis in a case of skin disease is sometimes a difficult object to attain ; to effect a cure is even more puzzling and annoying. Who has not had his professional vanity sadly tried by an obstinate case of tinea tonsurans, acne, or eczema, after running through the whole armamentarium of the pharmacopœia, only to find that it still persists? Having had ample opportunity of sitting at the feet of such Gamaliels and lions on skin as Jonathan Hutchison, Living, Malcom Morris, Sangster, and Stephen Mackenzie, and carefully noted their line of treatment, I have ventured to throw together in simple outline some remarks as to the best method of combatting the more common forms of these diseases. And, first, eczema : In acute eczema, the best local application is lotio plumbi applied on lint, the lint being kept continually moist. Dusting powders, such as oxide of zinc and starch, will also be found useful, and a lotion of carbolic acid (1 in 40) will relieve the itching.

*Chronic Eczema.*—Carbolic acid here, as in the acute stage, is one of the most useful remedies. It may be applied in the form of either a lotion or ointment x to xv gr. ad. ℥ i of the ointment. Thymol, highly recommended by Dr. Crocker, of London, in the strength of v to xx gr. ad. ℥ i, might be tried. Similar in effect to carbolic acid are the preparations of tar, which are the most serviceable of all external remedies. To obtain good results they should be handled with care; unless used at the proper time, and of proper strength, they serve only to irritate, and when this occurs they should be abandoned at once. Tar is of most benefit when the disease has reached the chronic stage. It should never be used in the acute. If there be much swelling and inflammation it likewise should be withheld. Ointments of varying strength are the most suitable means of applying tar, for in addition to the stimulating effect of the remedy an emollient effect is obtained. The ointment should not be too strong—from i to ii ʒ ad. ℥ i is usually sufficient. The two forms of tar commonly used are the pix liquida and oleum cadinum.

℞ Olei cadini.................................. ʒ iss,
Cerati simplici................................ i,
Olei amygdalæ amar........................ gtt. vi.
M. Ft. ungt.

This makes one of the most elegant tarry preparations. But there is another preparation of tar which, although known to the profession in this country, is not so well known as it deserves to be—I refer to the liquor carbonis detergens. It is a saturated alcoholic solution of coal tar, and made by Wright & Co., of London, and J. P. Remington, of Philadelphia, and may be had at Kenneth Campbell's. It is in great repute in England, and yields most beneficial results. The ointments which are most generally used in the treatment of chronic eczema in the London hospitals are the ungt. petrolei co., and the nitrate of mercury ointment. Both are excellent. The following is the formula for the ungt. petrolei co.:

℞ Liq. carbonis deterg.......................... ʒ ss,
Hyd. am. chlor.............................. gr. x,
Vaseline........................................ ℥ i.
M. Ft. ungt.

If the skin is greatly infiltrated, or the epidermis much thickened, solutions of potassa fusa are used with excellent results, v gr. ad. ℥, is usually sufficient. When the eczema consists of very chronic, dry small patches, the best treatment is to blister with acetum cantharides or the liq. epispasticus.

Professor Hebra's treatment will succeed sometimes when other treatment fails. It is of special service in chronic eczema of the leg. It consists in the application of sapo viridis, followed by the immediate use of an oily ointment. The ointment used in preference by him being the ungt. diacyli. A small lump of the soap, the size of a nut, is smeared upon a piece of flannel. This is to be applied directly to the patch of disease and rubbed firmly, and with moderate pressure, upon the skin until all traces of the soap disappear. The piece of flannel is now dipped into warm water and again applied in the same manner to the part, when an abundant lather will be found. More water is added from time to time until copious suds cover the skin, when with clean water the diseased surface is thoroughly washed off, freed from all signs of soap, and carefully dried with a soft cloth or towel. The rubbing should be kept up, in mild cases, from five to ten minutes, in severe to about twenty minutes. The first application should always be somewhat moderate, that too great a destruction of epidermis be not produced. The sensations of the patient will always serve as a guide to this point. The application is not painful, as might be supposed; but, on the contrary, agreeable, and relieves the itching; as a rule, it at once affords ease to the patient. The skin immediately after the washing presents a red and angry appearance, and is now ready for the ointment; this is spread on strips or pieces of soft flexible muslin. It is well not to make one large piece cover the whole, but it is preferable to have several pieces, in order that they may be the better adapted to the skin. The ointment should be spread thickly on the rags, finally the part should have outside cloths applied to prevent the oil from oozing through, and be bound by a bandage. The bandage is a matter of moment, for its application contributes materially to the success of the treatment. It is essential that the ointment be brought in close contact with the skin and kept in position. The entire operation should be repeated twice daily, morning and evening.

*Eczema of Hands.*—Hands should be protected from all irritating influences; they should be kept out of water, and free use of soap prohibited, exposure to heat also avoided. Rubber gloves will be found useful. In the majority of cases stimulating ointments most useful, as calomel or boracic ointment.

*Eczema of Nipple.*—Best treated with sapo viridis and ungt. diachyli. Application of nitrate of silver xx gr. ad. ℥ i highly spoken of by Living.

*Eczema of Beard.*—Crusts removed by oil and poultice, hair cut away or shaved off; apply ungt. petrolei co. In chronic stage use stimulating ointments.

*Eczema of Eyelids.*—In mild cases apply nitrate of mercury ointment; in severe cases pull out eyelashes, and touch the edges with solution of potassa in water, x gr. ad. ℥ i (McCaul Anderson). The alkali should be immediately neutralized with dilute acetic acid. Operation repeated every few days, after which nitrate of mercury ointment applied.

*Eczema of Leg.*—In cases of moist eczema the most successful treatment is that with sapo viridis and ungt. diachyli. The limb should be carefully bandaged, and when eczema is associated with varicose veins Dr. Martin's elastic bandage should be applied.

Squire, of London, recommends the glycerole of the subacetate of lead, xv to xxx gr. ad. ℥i, in these cases.

*Eczema Intertrigo.*—Dusting powders of oxide of zinc and starch with or without calomel used. Ungt. zinci one of the best applications. Parts should be seldom washed.

*Eczema of the Genitals.*—Sapo viridis and ungt. diachyli; in acute stages lotio nigra followed by ungt. zinci and calomel. Carbolic acid x gr. ad. ℥i, useful. Thymol also useful. Painting the part with tr. iodini sometimes serviceable.

*Eczema of Head.*—After the crusts have been removed by poulticing, the best application is the ungt. hydr. nit. In all cases of eczema the ordinary washing with soap and water must be forbidden, and this is especially the case when the delicate and healthy new cuticle is forming, for then water macerates and destroys it, and thus the duration of the disease is needlessly prolonged. While the local treatment is of paramount importance in eczema, the constitutional is not to be neglected; arsenic should be given and tonics of iron, quinine, etc., administered.

*Psoriasis.*—When psoriasis covers the whole trunk, or is nearly universal, the best treatment is by alkaline warm baths. Pot. carb. ℥ii to ℥iii should be added to an ordinary bath. The patient should remain in the bath for at least an hour and a half daily to do any good. The best time for taking the bath is shortly before going to bed, to avoid dressing again. The temperature of the bath should be 90° to 98°. After coming out of the bath the patient should be rubbed and anointed with vaseline, which should afterward be wiped off. When psoriasis attacks a leg, or a not too extensive surface of the body, then the tarry preparations and chrysophanic acid will be found most beneficial. Of the two I prefer the applicatian of tar; it may be applied either as an ointment or lotion, the latter most satisfactory; it dries quickly, and does not easily rub off on the clothes. My treatment would be to paint the liq. carb. deterg. with a camel's hair brush over the part affected two or three times a day. Chrysophanic acid has certainly yielded splendid results, and is much more active than tar; but it has a great many disadvantages, as setting up inflammation, staining the clothes and hair, etc. It is used in the strength of ʒi ad. ℥i of vaseline. Pyrogallic acid xxx gr. ad. ℥i is useful, and less open to the objections of the former.

In dealing with psoriasis of the scalp the free use of soap or the spirits of soap is very good, followed by the liq. carb. deterg. or the red or white precipitate ointment diluted with vaseline. In the chronic spots of psoriasis, about the knees, the same treatment is excellent. Obstinate cases of psoriasis often yield to the tinct. saponis. viridis c. pce., which consists of equal parts of pix liquida. alcohol and sapo viridis. Sulphuret of calcium has been highly recommended in these cases. The following is a good formula:

℞ Calcis, .......................................... ℥ss,

Sulphuris sublimati ............................ ℥i,

Aquæ ............................................. ℥x.

M. In the constitutional treatment arsenic and tonics should be given, and always remember the possibility of the gouty and scrofulous diathesis. I have seen cases of psoriasis rebellious to all local treatment, yield like a charm to vin. colchici. I need not say that the patients in these cases were gouty.

*Scabies.*—Sulphur ointment, half the strength of the Pharmacopœia ointment, is the remedy which you will find to do the most good. The best time to use it is at night. Give it with the following directions: To be rubbed all over the body, with the exception of the head and especially on the hands, buttock and lower part of the abdomen; and the underclothing used during the previous day, as socks, gloves, drawers and jersey should be worn during the night. This thoroughly disinfects the clothes, at the same time keeping the ointment well applied. In the morning a warm bath should be taken. The process should be repeated for three nights, and subsequently the ointment should be rubbed on the hands, wrists and buttocks for a few nights. When you are confronted with a case which you have had under treatment, but are not certain whether it is cured or not, you will find an ointment of bal. of Peru (ʒ ii ad. ℥ i) an excellent application It does not irritate or annoy the patient.

*Tinea Tonsurans.*—In mild cases painting the part with tr. iod., and afterwards apply an ointment made with hydr. ammon. xx gr. ad. ℥ i will be all that is necessary. In more severe cases the oleate of mercury ointment, 10 per cent. solution made by rubbing x gr. of freshly precipitated yellow oxide of mercury with xc gr. of oleic acid until dissolved, is one of the very best applications. A point of some moment, which I have often heard Dr. Living, of London, lay stress upon in his clinique, is to order the patient to have the head smeared over with carbolized glycerine in order to prevent the disease spreading to others. Dr. Alder Smith recommends equal parts of carbolic acid, citrine ointment and sulphur ointment as very effectual. If the disease is in an early stage, and consists of one or two circumscribed spots, the best plan is to cut the hair short all around the spots, and apply with brush Coster's paste, which consists of:

℞ Tr. iodii.................................... ʒ ii,

Ol. Picis.................................... ℥ i. M.

*Lichen.*—The remedy *par excellence* is arsenic internally—Fowler's solution most commonly used. Some soothing lotion should be used externally such as the appended :

℞ Sodæ biborate.............................. } aa ʒ ii,

Sodæ bicarb.............................. }

Acid hydrocyan. dil......................... ʒ i,

Glycerine.................................... ℥ ii,

Aquæ ad...................................... ℥ viss.

M. Hutchison says in lichen planus start with liq. sodæ arsenitis, but if it does not get better give liq. arsenicalis, or both combined.

*Acne.*—You will find the following treatment of acne to be the most satisfactory. The face should be steamed every night by holding it over a basin of hot water for a few minutes. The skin should be then well rubbed for five or six minutes with soap and flannel, or a soft nail brush may be used with advantage when the skin will bear it ; the soap should then be sponged off with warm water. When the face has been dried the following lotion should be applied, and allowed to dry and remain on all night :

℞ Sulphur precip . . . . . . . . . . . . ʒ ii,

Glycerine . . . . . . . . . . . . ʒ ii,

Spt. vini . . . . . . . . . . . . ℥ i,

Aquæ calcis . . . . . . . . . . . . } aa ℥ iii.

Aquæ rosæ . . . . . . . . . . . . }

M. In inveterate cases of acne the following will be found particularly serviceable :

℞ Sapo mollis . . . . . . . . . . . . ℥ j

Spt. rectificate . . . . . . . . . . . . ℥ iss,

Ol. levandulæ . . . . . . . . . . . . M xx,

Aquæ ad . . . . . . . . . . . . ℥ vi.

M. Ft. Lot.

The lotion should be applied with a piece of flannel and vigorously rubbed on the skin. It should be washed off and then the sulphured lotion applied.

In treating diseases of the skin one should always bear in mind the late Professor Hebra's admirable advice: whatever course be adopted, constancy and perseverance are of the utmost importance. He who is always changing his plan of treatment is sure not to attain his object so quickly as one who steadily and patiently applies whatever remedy seems best suited to his case.—*Canada Record.*

